# Blood urea nitrogen is independently associated with renal outcomes in Japanese patients with stage 3–5 chronic kidney disease: a prospective observational study

**DOI:** 10.1186/s12882-019-1306-1

**Published:** 2019-04-02

**Authors:** Makiko Seki, Masaru Nakayama, Teppei Sakoh, Ryota Yoshitomi, Akiko Fukui, Eisuke Katafuchi, Susumu Tsuda, Toshiaki Nakano, Kazuhiko Tsuruya, Takanari Kitazono

**Affiliations:** 1 Division of Nephrology and Clinical Research Institute, Department of Internal Medicine, National Hospital Organization Kyushu Medical Center, 1-8-1 Jigyohama, Chuo-ku, Fukuoka, 810-8563 Japan; 2 Department of Medicine and Clinical Science, Graduate School of Medical Sciences, Kyushu University, 3-1-1 Maidashi, Higashi-ku, Fukuoka, 812-8582 Japan; 3 Department of Nephrology, Nara Medical University, 840 Shijo-cho, Kashihara, Nara, 634-8521 Japan

**Keywords:** Blood urea nitrogen, Renal outcome, Chronic kidney disease, Serum osmolality, Uremic toxicity

## Abstract

**Background:**

Blood urea nitrogen (BUN) is one of the substances that affects the calculated serum osmolality (cSosm). A previous study demonstrated that BUN and cSosm were independently associated with the development of chronic kidney disease (CKD) in patients with preserved kidney function. In advanced CKD stages, there is a concomitant increase in cSosm and BUN levels. However, it remains unclear whether BUN or cSosm levels are related to renal outcomes in patients with moderate to severe kidney dysfunction. The aim of this study was to clarify whether the BUN or cSosm level is associated with kidney disease progression in patients with advanced CKD.

**Methods:**

In this prospective study, we enrolled 459 patients with CKD (stages 3–5). The composite renal endpoint was end-stage renal disease (ESRD) or death, and ESRD alone was added as an alternative outcome. A Cox proportional hazards model was utilized to determine the risk factors for a poor renal outcome. We adjusted for covariates including estimated glomerular filtration rate (eGFR). The cSosm (mOsm/kg) was calculated using the following formula: (2 × sodium) + (BUN/2.8) + (glucose/18).

**Results:**

During a median follow-up of 25.8 months, the renal endpoint was observed in 210 patients. Multivariable Cox analysis determined the hazard ratio (HR) [95% confidence interval (CI)] for the composite renal outcome in the second, third, and fourth BUN quartiles were 1.36 (0.72–2.58), 1.87 (0.95–3.66), and 2.66 (1.23–5.76) (*P* for trend < 0.01), respectively compared with the first BUN quartile. Conversely, by multivariable Cox analysis, the HRs (95% CIs) for poor outcomes in the second, third, and fourth cSosm quartiles, compared with the first cSosm quartile, were 1.13 (0.69–1.87), 0.95 (0.58–1.55), and 1.26 (0.78–2.03), respectively (*P* for trend = 0.39). In addition, with regard to the renal outcome of ESRD alone, higher BUN quartiles had a significantly increased risk for the outcome, but cSosm levels were not associated with the outcome.

**Conclusions:**

Higher BUN levels, but not cSosm levels, were associated with adverse renal outcomes independent of the eGFR, suggesting that BUN may be a useful marker for predicting kidney disease progression.

**Electronic supplementary material:**

The online version of this article (10.1186/s12882-019-1306-1) contains supplementary material, which is available to authorized users.

## Background

Urea is the primary metabolite derived from dietary protein and tissue protein turnover. It is freely filtered at the glomerulus but not secreted, and it is reabsorbed by the renal tubules. In addition, as urine flow rates decrease, more urea is reabsorbed [1]. Blood urea nitrogen (BUN) measures the nitrogen component of serum urea. BUN levels are inversely correlated with the decline of kidney function [2] and are also affected by extrarenal factors such as protein intake, gastrointestinal bleeding, catabolic states, malnutrition, heart failure, dehydration, use of glucocorticoids, and hepatic urea synthesis [3].

Under physiologic conditions urea slowly dissociates into cyanate, which is rapidly converted to isocyanate. Isocyanate is a reactive electrophile with high affinity for nucleophilic groups such as primary amines [4]. Carbamylation has been recognized to be a spontaneous post-translational modification of amino acids and proteins mediated by cyanate, leading to biochemical alterations. It has also been demonstrated that urea can exert direct toxicity on various tissues such as the intestinal epithelium, vascular walls, pancreatic β-cells and adipocytes, and indirect toxicity via carbamylation [5, 6]. Notably, BUN levels are associated with mortality among patients with heart failure [7, 8].

Experimental studies have demonstrated that the release of vasopressin and the activation of the aldose reductase-fructokinase pathway caused by the elevated serum osmolality might be associated with kidney injury [9, 10]. BUN is one of the substances that affects the calculated serum osmolality (cSosm) levels. Therefore, in patients in advanced stages of chronic kidney disease (CKD), there is a concomitant rise in cSosm and BUN levels. Furthermore, a recent study showed that higher BUN and cSosm levels were independently associated with the development of CKD, which was defined as a decrease in the estimated glomerular filtration rate (eGFR) to < 60 mL/min/1.73 m^2^ in patients with preserved kidney function (average eGFR of 86.7 mL/min/1.73 m^2^) [11]. However, it remains unclear whether high BUN or cSosm levels are an independent risk factor for kidney disease progression in patients with moderate to severe CKD. Thus, the aim of the present study was to determine whether the BUN or cSosm level is associated with adverse renal outcomes in patients with CKD (stages 3–5) independent of the eGFR. Additionally, a previous study demonstrated that eGFR and serum creatinine were associated with kidney disease progression in CKD patients [12]. Thus, we also evaluated whether BUN levels were related to CKD progression, independent of an alternative kidney function marker, serum creatinine.

## Methods

### Patients and study design

**Fig. 1:**
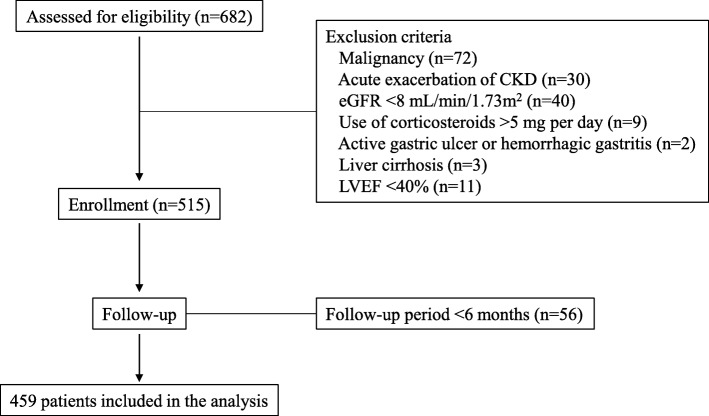
Flow chart of the enrollment process. CKD, chronic kidney disease; eGFR, estimated glomerular filtration rate; LVEF, left ventricular ejection fraction

Six hundred eighty-two consecutive Japanese patients with CKD stage 3–5 were admitted to our hospital, the National Hospital Organization Kyushu Medical Center, for evaluation and education about CKD between June 2009 and December 2017. Figure 1 shows a flow chart of the patient enrollment process. To decrease the effects of extrarenal factors on BUN levels, patients who used corticosteroids at a dose > 5 mg/day, or who had an active gastric ulcer or hemorrhagic gastritis, liver cirrhosis, or a left ventricular ejection fraction < 40% were excluded. Four hundred fifty-nine patients were analyzed. After discharge, patients were prospectively followed at our hospital for at least 6 months. Data were collected until July 2018. The composite renal endpoint was end-stage renal disease (ESRD) or death, whichever occurred first, and ESRD alone was added as an alternative outcome. ESRD was defined as renal dysfunction requiring renal replacement therapy such as maintenance hemodialysis or peritoneal dialysis or having undergone kidney transplantation. When the patients showed gradual worsening of kidney function during follow-up and were determined to require renal replacement therapy, they were diagnosed with ESRD and were re-admitted for renal replacement therapy. Time to ESRD was defined as the duration from baseline to the day of the first dialysis session or the kidney transplantation procedure. Death occurred before reaching ESRD. Information regarding patient death and definitions of censored patients are described in our previous report [13]. In total, 69 patients were censored; 63 were lost to follow-up and 6 required maintenance hemodialysis because of acute exacerbation of kidney function due to infectious diseases or congestive heart failure. As a result, 390 patients were completely followed up until July 2018.

### Clinical and biochemical assessments

Blood samples were obtained from each patient in the early morning after an overnight fast. Daily proteinuria was also measured. The eGFR (mL/min/1.73 m^2^) was calculated using the following new equation for Japanese patients: 194 × serum creatinine^− 1.094^ × age^− 0.287^ × 0.739 (if female) [14]. The cSosm (mOsm/kg) was calculated using the following formula: (2 × sodium) + (BUN/2.8) + (glucose/18) [15].

Clinical assessments at admission and the definitions of hypertension, diabetes mellitus, smoking, and dyslipidemia are described in our previous report [13]. An echocardiogram was performed to assess left atrial diameter and left ventricular ejection fraction.

### Statistical analysis

Continuous data are expressed as the mean ± SD or median (interquartile range) depending on their distribution. Participant BUN levels were divided into quartiles. Categorical data were compared across BUN quartiles using the chi-squared test with the Fisher’s exact test used for groups containing fewer than five individuals in any given cell. An analysis of variance (ANOVA) was used to compare continuous variables that were approximately normally distributed, and the Kruskal–Wallis test was used to compare skewed continuous variables. Participant cSosm levels were also divided into quartiles. Spearman’s rank correlation coefficients were used to identify the association between two continuous variables.

A Cox proportional hazards model was used to determine whether BUN or cSosm were associated with renal outcomes. Cox models were also used to assess the association of higher quartiles of each variable with renal outcomes with the lowest quartile serving as the reference category. Hazard ratios (HRs) and 95% confidence intervals (CIs) were calculated for each variable. Stratified analyses were also performed to calculate adjusted HRs for composite renal outcomes for every 10-mg/dL increase in BUN in subgroups stratified in accordance with baseline characteristics. Restricted cubic spline analyses were performed to qualitatively evaluate any nonlinear relationship between renal composite outcomes and BUN levels adjusted for age, sex, diabetes mellitus, smoking, dyslipidemia, systolic blood pressure, use of immunosuppressants and diuretics, body mass index, C-reactive protein, daily proteinuria, hemoglobin, eGFR, serum phosphorus, and serum albumin. We placed four knots at the 5th, 35th, 65th, and 95th percentiles of the BUN levels, and a median BUN value (18 mg/dL) in the reference group (first quartile) was selected as a reference for spline plots. Survival curves were estimated using the Kaplan–Meier method and evaluated using the log-rank test. Collinearity among covariates was assessed by measurement of the variance inflation factor (VIF) and tolerance value of each covariate. Furthermore, we examined the associations of cSosm levels with outcomes when removing one variable at a time in order of descending VIF. All statistical analyses were performed using STATA ver. 14 (Stata Corp., College Station, TX, USA). A *P-*value < 0.05 was considered to indicate statistical significance.

## Results

Table 1 shows the baseline clinical characteristics of patients according to quartiles of BUN. The median age of the 459 patients was 72.6 years (range, 30–94 years). The median eGFR for all participants was 22.6 mL/min/1.73 m^2^ (range, 8.1–59.3 mL/min/1.73 m^2^). Of the 459 patients, 161 (35%), 178 (39%), and 120 (26%) were categorized as CKD stage 3, 4, and 5, respectively. The primary causes of renal disease were chronic glomerulonephritis (21%, 95 patients), hypertensive nephrosclerosis (35%, 161 patients), diabetic nephropathy (27%, 124 patients), other defined causes (15%, 68 patients), and unknown (2%, 11 patients). The number of patients administered diuretics and AST-120 increased in the higher BUN quartiles. AST-120 is an oral intestinal spherical carbon adsorbent that has been reported to decrease serum indoxyl sulfate and ameliorate the decline in renal function [16]. As BUN levels increased, higher daily proteinuria and serum phosphorus levels and lower hemoglobin and eGFR levels were observed. With higher BUN levels, cSosm levels also increased. A significant difference was observed in left atrial diameter but not in left ventricular ejection fraction among BUN quartiles. In addition, cSosm positively correlated with BUN (r = 0.657, *P* < 0.01) and serum sodium (r = 0.541, *P* < 0.01) but not with fasting glucose (r = 0.008, *P* = 0.87).

**Table 1:** Baseline clinical characteristics of patients according to quartiles of BUN levels

Variables	All patients (*n* = 459)	BUN				*P*
		Q1 (*n* = 128)	Q2 (*n* = 104)	Q3 (*n* = 114)	Q4 (*n* = 113)	
		(8–23 mg/dL)	(24–30 mg/dL)	(31–41 mg/dL)	(42–94 mg/dL)	
Age (years), median (IQR)	72.6 (62.2–78.6)	66.6 (55.1–74.0)	73.6 (65.4–79.0)	73.8 (64.2–80.5)	74.9 (65.4–80.8)	< 0.01
Male, *n* (%)	320 (70)	85 (66)	73 (70)	73 (64)	89 (79)	0.07
Diabetes mellitus, *n* (%)	203 (44)	44 (34)	57 (55)	50 (44)	52 (46)	0.02
Hypertension, *n* (%)	427 (93)	107 (84)	99 (95)	112 (98)	109 (96)	< 0.01
Smoking, *n* (%)	256 (56)	68 (53)	57 (55)	61 (54)	70 (62)	0.49
Dyslipidemia, *n* (%)	343 (75)	99 (77)	81 (78)	85 (75)	78 (69)	0.41
Use of RAAS inhibitors, *n* (%)	357 (78)	102 (80)	80 (77)	93 (82)	82 (73)	0.39
Use of AST-120, *n* (%)	30 (7)	1 (1)	2 (2)	4 (4)	23 (20)	< 0.01
Use of diuretics, *n* (%)	163 (36)	26 (20)	40 (38)	46 (40)	51 (45)	< 0.01
SBP (mmHg), mean (SD)	137 (18)	134 (18)	134 (17)	137 (18)	137 (17)	0.34
DBP (mmHg), mean (SD)	72 (12)	74 (11)	71 (14)	73 (11)	69 (11)	0.01
BMI (kg/m^2^), median (IQR)	22.7 (20.9–25.4)	22.7 (20.6–25.5)	24.0 (21.6–26.3)	22.6 (21.0–25.4)	21.8 (19.7–24.5)	< 0.01
CRP (mg/dL), median (IQR)	0.09 (0.05–0.18)	0.08 (0.05–0.14)	0.12 (0.06–0.20)	0.08 (0.05–0.19)	0.08 (0.05–0.20)	0.05
Daily proteinuria (g/day), median (IQR)	1.10 (0.34–2.99)	0.74 (0.20–2.92)	0.93 (0.21–2.49)	1.52 (0.38–3.31)	1.36 (0.67–2.79)	0.01
Hemoglobin (g/dL), median (IQR)	10.4 (9.1–11.9)	12.1 (10.6–13.4)	10.8 (9.9–12.2)	10.1 (8.9–11.2)	9.0 (8.2–10.1)	< 0.01
eGFR (mL/min/1.73m^2^), median (IQR)	22.6 (14.7–35.0)	40.8 (33.7–50.4)	28.2 (22.5–33.5)	18.3 (14.1–22.2)	13.1 (10.2–15.8)	< 0.01
Serum albumin (g/dL), median (IQR)	3.4 (3.0–3.7)	3.5 (3.1–3.8)	3.5 (3.1–3.8)	3.4 (3.0–3.7)	3.3 (3.0–3.8)	0.22
Serum phosphorus (mg/dL), median (IQR)	3.7 (3.3–4.2)	3.4 (3.2–3.8)	3.5 (3.2–4.0)	3.8 (3.4–4.1)	4.2 (3.9–4.8)	< 0.01
cSosm (mOsm/kg), median (IQR)	297 (293–301)	293 (290–295)	296 (293–299)	298 (295–301)	302 (299–307)	< 0.01
LVEF (%), median (IQR)	68.4 (63.9–73.1)	68.8 (65.5–73.6)	69.3 (64.0–74.6)	67.6 (62.2–72.3)	68.1 (62.8–72.3)	0.09
LAD (mm), median (IQR)	41 (36–45)	39 (34–43)	42 (38–46)	41 (37–44)	41 (36–46)	< 0.01

Values are expressed as means ± SD, number (percent) or median (IQR). *BUN* blood urea nitrogen, *IQR* interquartile range, *RAAS* renin–angiotensin–aldosterone system, *SBP* systolic blood pressure, *DBP* diastolic blood pressure, *BMI* body mass index, *CRP* C-reactive protein, *eGFR* estimated glomerular filtration rate, *cSosm* calculated serum osmolality, *LVEF* left ventricular ejection fraction, *LAD* left atrial diameter

The median follow-up period was 25.8 months (range, 1.2–95.8 months). At the end of follow-up, 210 patients had reached the composite renal endpoint. Thirty-seven patients died before reaching ESRD. The causes of death were infectious disease in nine patients, sudden death in seven, uremia in five, malignancy in four, cardiac diseases in five, other defined causes in five, and unknown in two.

**Fig. 2:**
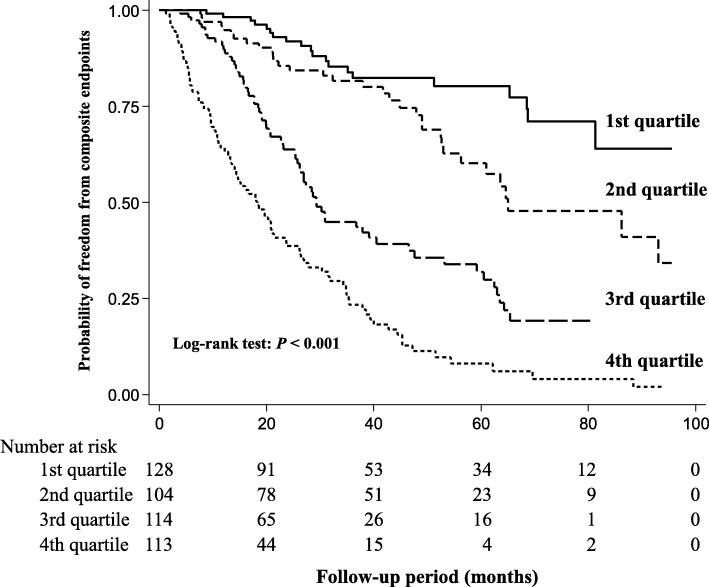
Kaplan–Meier curves for freedom from composite renal outcome in patients stratified by quartiles of BUN and compared using log-rank tests. BUN, blood urea nitrogen

**Fig. 3:**
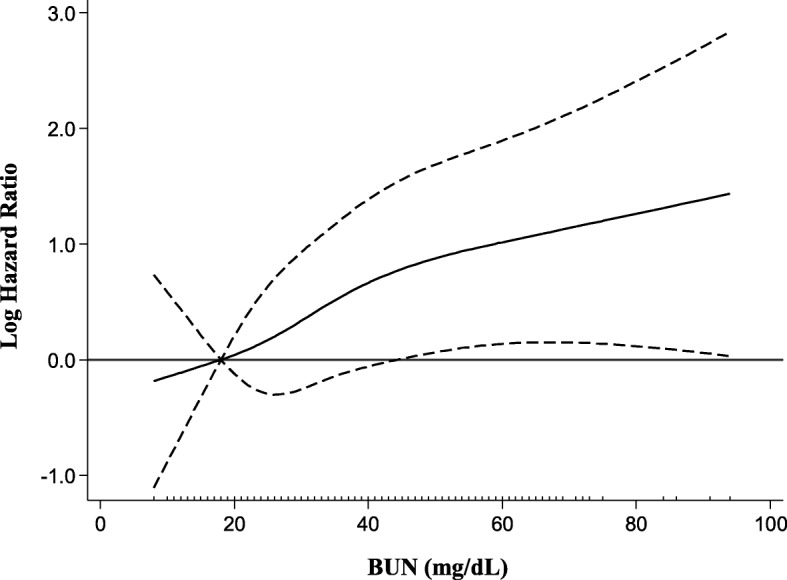
Adjusted hazard ratio for composite renal outcome according to BUN levels. The solid line indicates the hazard ratio and the dashed lines the 95% confidence interval. BUN, blood urea nitrogen

Kaplan–Meier analysis showed significantly higher rates of renal events in the higher BUN quartiles (Fig. 2). Table 2 shows the HRs for composite renal outcome according to quartiles of BUN or cSosm. In a multivariable analysis (Model 3), higher BUN quartiles had a significantly higher risk of adverse renal outcomes compared with the lowest quartile. In contrast, compared with the lowest cSosm quartile, no significant increase in the risk of poor renal outcomes in the higher quartiles of cSosm was found in Model 3. A spline analysis of the relationship between BUN and the risk of adverse renal outcomes showed that the risk of poor renal outcomes was progressively higher as the BUN level increased (Fig. 3).

**Table 2:** Hazard ratios for composite of ESRD or death associated with BUN and cSosm levels

		No. of events	^a^Incidence rate	Model 1			*P* for trend	Model 2			*P* for trend	Model 3			*P* for trend
				HR	95% CI	*P*		HR	95% CI	*P*		HR	95% CI	*P*	
BUN (mg/dL)															
8–23	Q1 (*n* = 128)	20	4.76	reference			< 0.01	reference			< 0.01	reference			< 0.01
24–30	Q2 (*n* = 104)	33	9.47	2.07	1.18–3.64	0.01		1.81	1.03–3.18	0.04		1.36	0.72–2.58	0.34	
31–41	Q3 (*n* = 114)	65	23.56	5.46	3.27–9.11	< 0.01		5.34	3.21–8.87	< 0.01		1.87	0.95–3.66	0.07	
42–94	Q4 (*n* = 113)	92	47.06	11.65	7.06–19.21	< 0.01		11.71	7.12–19.25	< 0.01		2.66	1.23–5.76	0.01	
cSosm (mOsm/kg)															
277–293	Q1 (*n* = 115)	29	7.44	reference			< 0.01	reference			< 0.01	reference			0.39
293–297	Q2 (*n* = 115)	41	13.45	1.86	1.15–3.00	0.01		1.60	0.99–2.60	0.05		1.13	0.69–1.87	0.62	
297–301	Q3 (*n* = 115)	52	16.32	2.23	1.40–3.54	< 0.01		1.82	1.14–2.90	0.01		0.95	0.58–1.55	0.85	
301–325	Q4 (*n* = 114)	88	39.18	5.58	3.64–8.56	< 0.01		4.79	3.11–7.38	< 0.01		1.26	0.78–2.03	0.35	

Model 1: adjusted for age and sex

Model 2: model 1 plus adjusted for diabetes mellitus, smoking, systolic blood pressure, and dyslipidemia

Model 3: model 2 plus adjusted for use of immunosuppressants and diuretics; C-reactive protein; body mass index; daily proteinuria; hemoglobin; eGFR; serum phosphorus; and serum albumin

*ESRD* end-stage renal disease, *BUN* blood urea nitrogen, *cSosm* calculated serum osmolality, *HR* hazard ratio, *CI* confidence interval, *eGFR* estimated glomerular filtration rate. ^a^Incidence rate per 100 person-years

**Fig. 4:**
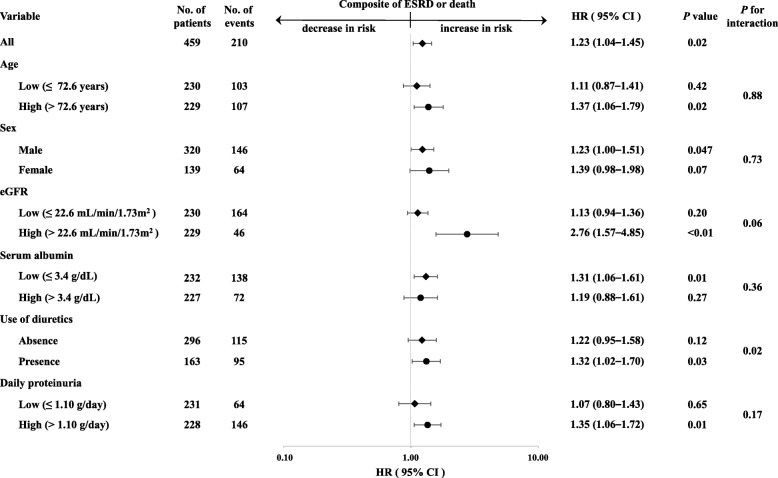
Adjusted hazard ratios and 95% confidence intervals for the composite renal outcome for every 10-mg/dL increase in BUN in subgroups stratified according to baseline characteristics. Adjusted for the same covariates as Model 3 in Table 2. ESRD, end-stage renal disease; BUN, blood urea nitrogen; eGFR, estimated glomerular filtration rate

Figure 4 summarizes adjusted HRs according to subgroups stratified by demographic and clinical characteristics. In all subjects, every 10-mg/dL increase in the BUN level was associated with an increased risk of adverse renal composite outcomes (HR, 1.23; 95% CI, 1.04–1.45). In male subjects, higher BUN levels were associated with a significant increase in the risk of adverse renal composite outcomes. In patients with a higher eGFR, lower serum albumin, and higher proteinuria level, the risk of poor renal outcomes significantly increased as the BUN level increased. In addition, in patients taking diuretics, as the BUN level increased, a significant increase in the risk of a poor renal outcome was found. No significant interactions for renal outcomes, except for use of diuretics (*P* = 0.02), were observed between BUN levels and other baseline clinical characteristics (*P* for interaction, 0.06–0.88).

We also investigated the association of BUN and cSosm levels with progression to ESRD (renal events, *n* = 173) (Table 3). In the fully adjusted model (Model 3), compared with the first quartile of BUN, the fourth quartile of BUN had a significantly increased risk of the composite outcome, whereas higher cSosm quartiles did not show a significant increase in the risk for poor outcomes.

**Table 3:** Hazard ratios for ESRD associated with BUN and cSosm levels

		No. of events	^a^Incidence rate	Model 1			*P* for trend	Model 2			*P* for trend	Model 3			*P* for trend
				HR	95% CI	*P*		HR	95% CI	*P*		HR	95% CI	*P*	
BUN (mg/dL)															
8–23	Q1 (*n* = 128)	15	3.61	reference			< 0.01	reference			< 0.01	reference			0.03
24–30	Q2 (*n* = 104)	29	8.35	2.72	1.46–5.11	< 0.01		2.25	1.20–4.24	0.01		1.45	0.71–2.97	0.31	
31–41	Q3 (*n* = 114)	58	21.20	7.24	4.06–12.92	< 0.01		6.74	3.79–11.98	< 0.01		1.75	0.83–3.71	0.14	
42–94	Q4 (*n* = 113)	71	37.62	14.59	8.18–26.03	< 0.01		14.64	8.24–26.01	< 0.01		2.42	1.03–5.67	0.04	
cSosm (mOsm/kg)															
277–293	Q1 (*n* = 115)	21	5.42	reference			< 0.01	reference			< 0.01	reference			0.07
293–297	Q2 (*n* = 115)	33	10.87	2.21	1.28–3.85	< 0.01		1.79	1.03–3.12	0.04		1.31	0.73–2.34	0.36	
297–301	Q3 (*n* = 115)	43	13.65	2.89	1.70–4.92	< 0.01		2.12	1.24–3.61	< 0.01		0.96	0.55–1.70	0.90	
301–325	Q4 (*n* = 114)	76	34.72	7.56	4.61–12.38	< 0.01		6.06	3.69–9.96	< 0.01		1.64	0.95–2.82	0.07	

Model 1: adjusted for age and sex

Model 2: model 1 plus adjusted for diabetes mellitus, smoking, systolic blood pressure, and dyslipidemia

Model 3: model 2 plus adjusted for use of immunosuppressants, and diuretics; daily proteinuria; hemoglobin; eGFR; serum phosphorus; and serum albumin

*ESRD* end-stage renal disease, *BUN* blood urea nitrogen, *cSosm* calculated serum osmolality, *HR* hazard ratio, *CI* confidence interval, *eGFR* estimated glomerular filtration rate. ^a^Incidence rate per 100 person-years

The collinearity among the variables used in Model 3 was assessed, as shown in Additional file 1: Table S1 (for composite outcomes) and Additional file 2: Table S2 (for ERSD alone). All variables had low VIF values. Furthermore, Additional file 3: Table S3 (for composite outcomes) and Additional file 4: Table S4 (for ESRD alone) show the adjusted HRs for outcomes after the removal of one variable at a time. In both analyses, the removal of three variables (daily proteinuria, serum albumin, and hemoglobin) did not result in a significant association between higher cSosm levels and outcomes. A significant association was first shown by the removal of the fourth variable (eGFR).

### Sensitivity analysis

To assess the robustness of the findings shown in Table 2, we performed sensitivity analysis, and these results are shown in Table 4. In a multivariable analysis, we included serum creatinine as a covariate, instead of eGFR. Higher BUN quartiles, but not cSosm quartiles, were independently associated with adverse renal composite outcomes. These findings were similar to those shown in Table 2.

**Table 4:** Hazard ratios for composite of ESRD or death associated with BUN and cSosm levels (sensitivity analysis)

	Analysis 1			
	HR	95% CI	*P*	*P* for trend
BUN				
Q1	reference			< 0.01
Q2	1.86	1.01–3.40	0.045	
Q3	2.66	1.44–4.89	< 0.01	
Q4	3.11	1.50–6.46	< 0.01	
cSosm				
Q1	reference			0.42
Q2	1.12	0.68–1.85	0.66	
Q3	1.03	0.63–1.69	0.90	
Q4	1.24	0.75–2.04	0.40	

Analysis 1: adjusted for age; sex; diabetes mellitus; smoking; systolic blood pressure; dyslipidemia; use of immunosuppressants, and diuretics; C-reactive protein; body mass index; daily proteinuria; hemoglobin; serum creatinine; serum phosphorus; and serum albumin

*ESRD* end-stage renal disease, *BUN* blood urea nitrogen, *cSosm* calculated serum osmolality, *HR* hazard ratio, *CI* confidence interval

## Discussion

The present study demonstrated that higher BUN levels were associated with adverse renal outcomes independent of eGFR in patients with moderate to severe CKD. A recent study reported that both BUN and cSosm were independent risk factors for CKD in patients with preserved kidney function [11]. However, in the present study, a multivariable Cox analysis (Model 3) did not show a significant association between cSosm and poor renal outcomes. These findings suggest that the absolute BUN, rather than serum osmolality, could have predictive value for kidney disease progression. Conversely, in our cohort, higher BUN levels were significantly associated with poor outcomes in the higher but not the lower eGFR group, although there was no significant interaction for renal outcomes between BUN and eGFR levels as shown in Fig. 4. These results might suggest that the impact of the BUN level on kidney disease progression is stronger at earlier stages of CKD.

Additionally, sensitivity analysis was performed, as shown in Table 4. The results of this analysis were similar to those observed in Table 2. These findings confirmed the robustness of our results. Thus, BUN levels may be associated with renal outcomes, independent of kidney function.

The pathophysiological mechanism underlying the association between BUN and adverse renal outcomes remains unclear. However, several hypotheses regarding the association have been considered. In CKD, declining kidney function, which is characterized by chronic elevation of BUN, is a pathological state that promotes the formation of isocyanate [17]. In fact, it was reported that the average plasma concentration of cyanate, which was 45 nmol/L in healthy individuals, increased up to 141 nmol/L in patients before dialysis [18]. Accordingly, when kidney function declines accompanied by the accumulation of urea, the burden of carbamylation naturally increases. Berg et al. demonstrated that the concentration of carbamylated albumin was approximately 2-fold higher in patients with both CKD (stages 3 and 4) and ESRD than in nonuremic patients, and that the positive correlation between BUN and carbamylated albumin was stronger in patients with CKD who were not on dialysis than in those with ESRD. Based on these results, they suggested that “hypercarbamylation” is present throughout the stages of CKD before and after initiation of hemodialysis [19]. Furthermore, it has been reported that carbamylated proteins are associated with all-cause and cardiovascular mortality in ESRD patients [19–21]. Notably, it was reported that carbamylated proteins caused tubular cell damage and peritubular fibrosis in the amphibian kidney [22] and enhanced mesangial cell proliferation as well as synthesis of collagen type I and IV in mesangial cells [23]. Unfortunately, in the present study, we could not examine carbamylated proteins. Nevertheless, given these findings, it could be speculated that the increased risk of adverse renal outcomes associated with higher BUN levels in the present study is attributable to the indirect toxicity of urea via carbamylation.

Urea at concentrations relevant to CKD has been reported to directly increase reactive oxygen species (ROS) and oxidative stress in adipocytes [24], pancreatic β–cells [25], and aortic endothelial cells [26]. D’Apolito et al. demonstrated that urea induced ROS production in adipocytes, leading to insulin resistance [24]. Koppe et al. also reported that urea increased oxidative stress and protein *O*-linked N-glucosamine acylation in islets, resulting in insulin secretory defects [25]. Recently, in a large cohort study, higher BUN levels were associated with an increased risk of diabetes mellitus; the HR (95% CI) for poor outcomes in patients with a BUN > 25 mg/dL was 1.23 (1.21–1.25) compared with a BUN ≤25 mg/dL [27]. These findings could support the above experimental findings by D’Apolito et al. [24] and Koppe et al. [25]. Alternatively, oxidative stress is progressively enhanced and correlates with the degree of kidney dysfunction in patients with CKD [28, 29]. Previous reports suggested that oxidative stress might be associated with the pathogenesis and progression of kidney disease. Urinary 8-hydroxydeoxyguanosine is considered a useful marker for predicting the development of diabetic nephropathy in diabetic patients [30]. It was also suggested that malondialdehyde might play an important role in the pathogenesis of glomerulosclerosis [31] and that advanced oxidation protein products were associated with the pathogenesis and progression of IgA nephropathy [32]. However, it remains unknown whether urea-induced oxidative stress directly contributes to kidney disease progression.

Urea production is directly proportional to the daily protein intake, and restriction of dietary protein intake results in a reduction of urea generation [33]. Many studies regarding the efficacy of dietary protein restriction on kidney disease progression have been conducted; however, results have been inconsistent. The largest randomized controlled trial, the Modification of Diet in Renal Disease study, demonstrated that a very low-protein diet (VLPD) supplemented with ketoacids slowed the rate of CKD progression compared with a low-protein diet (LPD), but this was not statistically significant [34]. Two meta-analyses in the 2000s noted that an LPD significantly decreased the risk of adverse renal outcomes in nondiabetic patients [35] but not in diabetic patients [36]. A recent meta-analysis investigated the effects of LPD and VLPD on various outcome measures in CKD patients [37]. Patients who received an LPD had a significantly reduced risk of progression to ESRD compared with those who received higher protein diets. In addition, the risk of progression to ESRD was significantly lower in patients who received a VLPD than in those who received an LPD. Garneata et al. also noted that a vegetarian VLPD supplemented with ketoanalogues could delay the need to initiate dialysis compared with an LPD and that VLPD recipients showed improvement in metabolic parameters, including a decrease in serum urea, compared with LPD recipients [38]. In the present study, we did not assess dietary protein intake or BUN levels in each patient after discharge. Therefore, we could not evaluate whether the amount of protein intake after discharge affected changes in BUN levels or adverse renal outcomes or whether the degree of change in BUN levels was related to the rate of CKD progression.

### Study limitations

This study had some limitations. First, patients who were admitted to a single regional hospital were recruited in this cohort; as a result, this study had relatively small sample size, old patients, and the number of male patients more than twice that of female patients. Accordingly, selection bias may exist in this population. In the stratified analyses (Fig. 4), higher BUN levels were independently associated with adverse renal outcomes in male subjects, but not in female subjects. In addition, a significant association was observed in the higher age group but not in the lower age group. Second, we did not examine uremic toxins, such as indoxyl sulfate and *p*-cresyl sulfate, which are associated with kidney disease progression [39, 40]. Third, we did not evaluate protein intake before admission or precise volume status at admission, which could have affected baseline BUN levels. A previous report suggested that left atrial diameter could be considered a marker of volume status in dialysis patients [41]. We assessed left atrial diameter in each patient and it was slightly increased in the higher BUN quartiles (Table 1). This suggests that higher BUN levels did not reflect volume depletion. Fourth, the follow-up period in the present study was relatively short. Finally, it might be considered that a single BUN measurement does not provide sufficient accuracy for predicting the renal outcome.

## Conclusions

Higher BUN levels were identified as a risk factor for kidney disease progression in patients with moderate to severe CKD, independent of eGFR. Although cSosm levels increased together with BUN levels, they were not independently associated with a poor outcome. Additionally, based on the sensitivity analysis, it is also suggested that BUN levels are associated with adverse renal outcomes, independent of kidney function. Our findings suggest that the measurement of BUN levels may be useful for predicting renal outcomes.

## Additional files

Additional file 1: **Table S1.** VIF and tolerance values of the variables in Model 3 (for composite outcomes). (DOCX 17 kb)

Additional file 2: **Table S2.** VIF and tolerance values of the variables in Model 3 (for ESRD alone). (DOCX 16 kb)

Additional file 3: **Table S3.** Hazard ratios for the association between the composite of ESRD or death and cSosm levels. (DOCX 17 kb)

Additional file 4: **Table S4.** Hazard ratios for the association between ESRD alone and cSosm levels. (DOCX 15 kb)

## Abbreviations

BUN: Blood urea nitrogen
CI: Confidence interval
CKD: Chronic kidney disease
cSosm: calculated serum osmolality
eGFR: estimated glomerular filtration rate
ESRD: End-stage renal disease
HR: Hazard ratio
LPD: Low-protein diet
VIF: Variance inflation factor
VLPD: Very low-protein diet
