# Supplementary material for: Blood urea nitrogen is independently associated with renal outcomes in Japanese patients with stage 3–5 chronic kidney disease: a prospective observational study
Source: BMC Nephrol. 2019 Apr 2;20:115. doi: 10.1186/s12882-019-1306-1 (PMC6444850; doi:10.1186/s12882-019-1306-1)
Supplement: Supplementary file 1 — Table S1. VIF and tolerance values of the variables in Model 3 (for composite outcomes). (DOCX 17 kb) [file 12882_2019_1306_MOESM1_ESM.docx]

**Additional file 1: Table S1.** VIF and tolerance values of the variables in Model 3 (for composite outcomes)

| Variables | VIF | Tolerance value |
| --- | --- | --- |
| Age | 1.48 | 0.68 |
| Sex | 1.58 | 0.63 |
| Diabetes mellitus | 1.31 | 0.76 |
| Smoking | 1.42 | 0.71 |
| Systolic blood pressure | 1.22 | 0.82 |
| Dyslipidemia | 1.14 | 0.87 |
| Use of immunosuppressants* | 1.20 | 0.83 |
| Use of diuretics* | 1.14 | 0.88 |
| C-reactive protein* | 1.09 | 0.92 |
| Body mass index* | 1.36 | 0.74 |
| Daily proteinuria* | 2.83 | 0.35 |
| Hemoglobin* | 2.34 | 0.43 |
| eGFR* | 1.97 | 0.51 |
| Serum phosphorus* | 1.54 | 0.65 |
| Serum albumin* | 2.56 | 0.39 |

*Indicates variables added in Model 3.

VIF, variance inflation factor; eGFR, estimated glomerular filtration rate.
