# Supplementary material for: Blood urea nitrogen is independently associated with renal outcomes in Japanese patients with stage 3–5 chronic kidney disease: a prospective observational study
Source: BMC Nephrol. 2019 Apr 2;20:115. doi: 10.1186/s12882-019-1306-1 (PMC6444850; doi:10.1186/s12882-019-1306-1)
Supplement: Supplementary file 2 — Table S2. VIF and tolerance values of the variables in Model 3 (for ESRD alone). (DOCX 16 kb) [file 12882_2019_1306_MOESM2_ESM.docx]

**Additional file 2: Table S2.** VIF and tolerance values of the variables in Model 3 (for ESRD alone)

| Variables | VIF | Tolerance value |
| --- | --- | --- |
| Age | 1.44 | 0.69 |
| Sex | 1.56 | 0.64 |
| Diabetes mellitus | 1.29 | 0.78 |
| Smoking | 1.41 | 0.71 |
| Systolic blood pressure | 1.22 | 0.82 |
| Dyslipidemia | 1.08 | 0.92 |
| Use of immunosuppressants* | 1.20 | 0.83 |
| Use of diuretics* | 1.12 | 0.90 |
| Daily proteinuria* | 2.78 | 0.36 |
| Hemoglobin* | 2.13 | 0.47 |
| eGFR* | 1.92 | 0.52 |
| Serum phosphorus* | 1.54 | 0.65 |
| Serum albumin* | 2.52 | 0.40 |

*Indicates variables added in Model 3.

VIF, variance inflation factor; eGFR, estimated glomerular filtration rate.
