# Supplementary material for: Blood urea nitrogen is independently associated with renal outcomes in Japanese patients with stage 3–5 chronic kidney disease: a prospective observational study
Source: BMC Nephrol. 2019 Apr 2;20:115. doi: 10.1186/s12882-019-1306-1 (PMC6444850; doi:10.1186/s12882-019-1306-1)
Supplement: Supplementary file 3 — Table S3. Hazard ratios for the association between the composite of ESRD or death and cSosm levels. (DOCX 17 kb) [file 12882_2019_1306_MOESM3_ESM.docx]

**Additional file 3: Table S3.** Hazard ratios for the association between the composite of ESRD or death and cSosm levels.

|  | Model A | | Model B | | Model C | | Model D | |
| --- | --- | --- | --- | --- | --- | --- | --- | --- |
| cSosm | HR | 95% CI | HR | 95% CI | HR | 95% CI | HR | 95% CI |
| Q1 | reference | | reference | | reference | | reference | |
| Q2 | 1.15 | 0.70–1.92 | 1.39 | 0.85–2.28 | 1.32 | 0.80–2.16 | 1.56 | 0.96–2.54 |
| Q3 | 0.98 | 0.60–1.60 | 0.96 | 0.59–1.55 | 0.97 | 0.60–1.57 | 1.46 | 0.91–2.34 |
| Q4 | 1.31 | 0.81–2.12 | 1.23 | 0.76–1.98 | 1.30 | 0.80–2.10 | 2.64 | 1.65–4.23 |

Model A: Adjusted for variables in Model 3 for composite outcomes (age, sex, diabetes mellitus, smoking, systolic blood pressure, dyslipidemia, use of immunosuppressants, use of diuretics, C-reactive protein, body mass index, daily proteinuria, hemoglobin, eGFR, serum phosphorus, and serum albumin) minus daily proteinuria.

Model B: Model A minus serum albumin.

Model C: Model B minus hemoglobin.

Model D: Model C minus eGFR.

ESRD, end-stage renal disease; cSosm, calculated serum osmolality; HR, hazard ratio; CI, confidence interval; eGFR, estimated glomerular filtration rate.
