# Supplementary material for: Blood urea nitrogen is independently associated with renal outcomes in Japanese patients with stage 3–5 chronic kidney disease: a prospective observational study
Source: BMC Nephrol. 2019 Apr 2;20:115. doi: 10.1186/s12882-019-1306-1 (PMC6444850; doi:10.1186/s12882-019-1306-1)
Supplement: Supplementary file 4 — Table S4. Hazard ratios for the association between ESRD alone and cSosm levels. (DOCX 15 kb) [file 12882_2019_1306_MOESM4_ESM.docx]

**Additional file 4: Table S4.** Hazard ratios for the association between ESRD alone and cSosm levels.

|  | Model A | | Model B | | Model C | | Model D | |
| --- | --- | --- | --- | --- | --- | --- | --- | --- |
| cSosm | HR | 95% CI | HR | 95% CI | HR | 95% CI | HR | 95% CI |
| Q1 | reference | | reference | | reference | | reference | |
| Q2 | 1.33 | 0.74–2.38 | 1.62 | 0.91–2.87 | 1.50 | 0.85–2.65 | 1.76 | 1.01–3.08 |
| Q3 | 0.99 | 0.56–1.75 | 0.99 | 0.57–1.73 | 0.99 | 0.57–1.73 | 1.63 | 0.95–2.81 |
| Q4 | 1.70 | 0.99–2.92 | 1.53 | 0.89–2.62 | 1.63 | 0.95–2.81 | 3.56 | 2.09–6.06 |

Model A: Adjusted for variables in Model 3 for ESRD alone (age, sex, diabetes mellitus, smoking, systolic blood pressure, dyslipidemia, use of immunosuppressants, use of diuretics, daily proteinuria, hemoglobin, eGFR, serum phosphorus, and serum albumin) minus daily proteinuria.

Model B: Model A minus serum albumin.

Model C: Model B minus hemoglobin.

Model D: Model C minus eGFR.

ESRD, end-stage renal disease; cSosm, calculated serum osmolality; HR, hazard ratio; CI, confidence interval; eGFR, estimated glomerular filtration rate.
